# Method matters: Use of thermal‐imaging drones to assess the assumptions of density estimation techniques

**DOI:** 10.1002/eap.70164

**Published:** 2025-12-08

**Authors:** David M. Delaney, Tyler M. Harms, Stephen J. Dinsmore

**Affiliations:** ^1^ Department of Natural Resource Ecology and Management Iowa State University Ames Iowa USA; ^2^ Iowa Department of Natural Resources Boone Research Station Boone Iowa USA

**Keywords:** abundance, distance sampling, mammal, N‐mixture, population size, study design, ungulate, white‐tailed deer

## Abstract

Techniques to estimate the density of unmarked animals are widely used by ecologists, but accurate estimates from these methods rely on assumptions about the study system. We conducted thermal‐imaging drone surveys to test the validity of three assumptions for conducting distance sampling on white‐tailed deer (*Odocoileus virginianus*) via nocturnal spotlight surveys in Iowa, USA. We found that the proportion of the population that occurred within forests that are unsamplable (i.e., availability bias) was negligible when vegetative green‐up was sparse but increased to more than 50% as spring green‐up progressed. The proportion of deer that were bedded, which are less detectable than standing or walking deer, depended on the day of year and time of night, suggesting these variables should be modeled on detection probability to reduce bias in parameter estimates. Lastly, we found evidence of road avoidance which influences how we analyze distance sampling data from road‐based survey designs. Each of these deviations from the assumptions of conventional distance sampling informs future sampling design and analysis and will improve the accuracy of density estimates in our system. More generally, our study provides an example of how drone surveys can be conducted to improve density estimation techniques for a range of animal systems.

## INTRODUCTION

The density of animals in a population is important to estimate for ecological studies and natural resource management (Callaghan et al., 2024; Nichols, 2014). Methods that estimate density from unmarked populations are often preferred to more intensive methods, such as mark‐recapture, because effort can be spread over broader spatial distributions (Seber, 1986). For example, many natural resource agencies need to annually estimate population size at county or management unit levels statewide (Rabe et al., 2002). To improve the accuracy of density estimates, methods estimate detection probability either from temporally replicated surveys (N‐mixture modeling design, Madsen & Royle, 2023; Royle, 2004) or from the spatial distribution of observed animals (distance sampling, Buckland et al., 2001; Burnham et al., 1980; see also White, 2005). Yet, these methods require assumptions about the study system to be met to produce unbiased estimates (discussed below), and robust assessment of these assumptions has been posited as a fruitful area of advancement in abundance estimation techniques (Elphick, 2008; Forsyth et al., 2022; Link et al., 2018).

Unmanned aerial vehicles (hereafter, drones) have become powerful tools for wildlife research because they provide an aerial view, often with the aid of thermal imaging and high‐resolution cameras, without the human risk or economic cost of manned aircraft (Christie et al., 2016; Corcoran et al., 2021; Iglay et al., 2024). While drones have been used for a variety of research purposes, including assessment of habitat (Barnas et al., 2019), demography (Schofield et al., 2017), and antipredator response (Schad & Fischer, 2022), hazing (Duttenhefner et al., 2024), and collection of biological samples (Geoghegan et al., 2018), they have most often been used to count wildlife (reviewed in Iglay et al., 2024; Linchant et al., 2015; Mo & Bonatakis, 2022). Drone surveys typically detect more animals than ground‐based surveys (Fettermann et al., 2022; Hodgson et al., 2016, 2018; McCarthy et al., 2022; Preston et al., 2021; but see Gentle et al., 2018; see also Saunders et al., 2022) but are limited in the area that can be studied by battery life, equipment costs, and pilot availability. As is typical with emerging technologies, the diversity of ways we can use drones to study wildlife continues to expand (Wirsing et al., 2022). A relatively untapped application of drones is testing the system‐specific assumptions of N‐mixture and distance sampling study designs. Validation of these assumptions should increase confidence in estimates and quantification of any bias can be used to correct population estimates. Below we discuss three major assumptions of N‐mixture and distance sampling techniques that drone surveys are well poised to evaluate.

The first assumption that drones are well suited to test is that the entire population of animals within the study area is available to sample (Kéry & Schmidt, 2008; Pollock et al., 2004). N‐mixture and distance sampling techniques estimate the probability that an individual is detected given that it is available to sample. Animals that are in locations inaccessible to observers (e.g., animals inside their burrows) are not sampleable, and density will be underestimated by the proportion of the population unavailable for sampling. Drones have been used to follow individual marine mammals to quantify the amount of time spent at depths that preclude detection by human observers (Brown et al., 2023; Hodgson et al., 2017). This proportion of time represents availability bias and can be used as a correction factor to improve the accuracy of density estimates. Aside from marine mammals, many terrestrial (Bushaw et al., 2019; Shirley & Janke, 2021) or semiaquatic (Bushaw et al., 2020) species exist in complex vegetation that precludes human observation from ground level, but can be observed with drones from above.

The second assumption that drones are well suited to test is that covariates which affect detection probability are known and modeled (Duarte et al., 2018; Link et al., 2018; Veech et al., 2016). For example, even among the available animals, individuals tend to be more detectable when they are actively moving (Jacques et al., 2014; Zabransky et al., 2016). Drone surveys that can more effectively identify potential individual‐, observation‐, or site‐level covariates likely to affect detection could inform model structure. Assessment of potential factors from ground‐based surveys alone may yield biased understandings because ground‐based surveys often disturb animals at the moment of, or just prior to, detection (Glennie et al., 2020). After experimental discovery of flight altitudes that do not disturb focal animals (Schroeder et al., 2020), drones may observe animals with less risk of disturbance than ground‐based observation (McCarthy et al., 2022).

Third, these methodologies assume animals are randomly distributed from observers (Buckland et al., 2001; Burnham et al., 1980; Miller et al., 2019). This is best assured by randomly choosing the locations of points or lines to conduct surveys, but studies often use roads or select other convenient landscape features to implement surveys (Buckland et al., 2015, p. 223; Collier et al., 2013; Marques et al., 2010). If animal density is different around chosen features then density estimates may not be representative of a broader area of interest. Furthermore, in distance sampling, detection probability is estimated from the decline (or lack thereof) in animal detections with distance from the observer (Buckland et al., 2001; Miller et al., 2019). Thus, either attraction to, or avoidance of, landscape features used as observation points will confound the estimation of detection probability. Despite concern over non‐uniform animal distributions from roads (Collier et al., 2013), road‐based wildlife surveys remain widely implemented because of their tradeoff of effort with presumed accuracy (Amos et al., 2014). Drone surveys could quantify the distribution of animals from roads which could be implemented into distance sampling model structure (sensu Delaney et al., 2025; Marques et al., 2013) or used to more accurately extrapolate density to areas farther from roads.

### Case study

The goal of this study was to explore how to use emerging drone technologies to robustly assess the assumptions of ground‐based density estimation techniques. Each spring, the Iowa Department of Natural Resources (DNR) conducts nocturnal spotlight surveys in each of Iowa's 99 counties to estimate the population dynamics of white‐tailed deer (*Odocoileus virginianus*; Kaminski et al., 2019). These data have been analyzed in a distance sampling framework to estimate county‐level densities (Dinsmore et al., 2024). We conducted a thermal‐imaging drone study to test three assumptions of this system to improve the annual density estimation of white‐tailed deer in Iowa, USA.

#### Assumption 1

All deer are within open habitat and are therefore available to sample during the DNR's nocturnal spotlight surveys. Surveys are conducted from 15 March to 27 May and begin 1 h after sunset and continue no later than 1 h before sunrise. At the beginning of this annual period, vegetative green‐up has not begun and deer are likely at their most food‐limited period of the year. Staff are encouraged to sample early in the year, prior to green‐up but constraints on staff time and climatic variability can lead to samples occurring after green‐up. Temporally replicated surveys during this time period (13 March–23 May) revealed lower deer counts later in the spring, possibly because a portion of the population remains in forest or other cover during nighttime samples instead of feeding in the open (Delaney et al., 2025b). To test this possibility, we flew a drone over a mix of forest and open landcovers during 12 nights across the beginning of vegetative green‐up in 2024.

#### Assumption 2

Deer behavior that could affect detectability is constant throughout the night and over the window of sampling dates. Deer that are standing, because they are moving or feeding, are likely more detectable than deer that are bedded (e.g., Jacques et al., 2014; Zabransky et al., 2016). We assessed whether the proportion of deer that are bedded changes by day of year or time of night.

#### Assumption 3

Deer are randomly distributed from transects, which occur along gravel roads. Gravel roads are used for the DNR's spotlight survey because traffic is less relative to paved roads. Prior studies (Giudice et al., 2019; McShea et al., 2011) and our exploratory analysis (Appendix S1: Figure S1) suggest deer avoid roads. Because the detection ability of observers from vehicles covaries with road avoidance behavior, we cannot quantify the magnitude of road avoidance with the data on hand. Therefore, we flew a drone adjacent to gravel roads and quantified the distribution of deer from above.

## METHODS

We used a rotary‐wing drone with an integrated thermal‐imaging camera (DJI Matrice 30T) to measure deer landscape use and behavior adjacent to gravel roads in Iowa. This drone weighed 3770 g, had dimensions of 470 × 585 × 215 mm (length × width × height), and could fly for 41 min per battery charge. The thermal camera had a resolution of 1280 × 1024 and each pixel captured approximately 15 cm of width at ground level. We conducted 18 h of flights on 12 nights from 9 to 30 April 2024. Flights occurred at least 1.5 h after sunset and finished no later than 1 h prior to sunrise; 95% of flights began between 21:25 and 01:25 h. Sites were sampled for an average of 26 min (range = 7–57 min). We only sampled on nights with winds <24 km/h and temperature >0°C (sensu the DNR's spotlight survey protocol).

### Sampling locations

We selected sampling locations by visually searching aerial photography of public and private properties adjacent to gravel roads (to resemble transects used for the DNR's spring spotlight survey) that contained a mix of forested and open landcovers. We only surveyed sites that landowners granted permission for and were therefore unable to randomly choose sites. Open landcover consisted of corn and soybean fields, hay/pasture, prairie, and grass. Forest landcover consisted mostly of deciduous woodlands with few coniferous trees. Early in the sampling window (beginning 9 April), vegetative leaf out was negligible, but increased during the study. We ended the study on 30 April because we were concerned that further leaf out could entirely block thermal signatures of deer below trees (and bias results). We sampled 41 sites (contiguous parcels) ranging from 17 to 316 ha (mean = 94, SD = 67; total area sampled = 3095 ha).

We visited each sampling location during daytime to verify site characteristics, search for flight hazards, and program the flight path using the remote control. All flights were video recorded and later processed to determine deer locations and behavior; see Delaney, Harms, and Dinsmore (2025) for two example videos recorded by the drone: https://doi.org/10.25380/iastate.30117049.v1. We flew the drone at a height of 100 m above ground and tilted the camera 30 degrees forward of straight down. This angle enabled differentiation of the legs, neck, and head of deer, which facilitated confidence in identifications. A straight‐down camera angle was more difficult to identify deer from oval rocks and smaller mammals. Deer did not appear disturbed by drone flight at 100 m above ground. Field of view was slightly wider than 100 m at the bottom of the image and ~120 m wide at the top of the image, farther from the drone. We designed flight paths in a grid pattern so that every point in the study area was within ~50 m horizontally of the drone; this was achieved by spacing parallel flight paths approximately 100 m apart (Appendix S1: Figure S2). Deer that were observed during more than one flight path were recorded as occurring at the first location they were detected. Flight speeds ranged from 1.2 to 2.1 m per second depending on wind speed; images were clearer on calm nights and therefore we could fly faster and cover more area during low wind nights. These flight parameters gave high confidence in deer identification and we believe we only failed to detect deer if they occurred beneath a rare thermal barrier, such as a coniferous tree or a leafed‐out deciduous tree that increased in frequency as spring progressed.

### Video processing

We watched video footage and geotagged still‐frame photos on a desktop computer. The location of each deer was determined based on geotagged video and photo location and comparison with aerial photography in ArcGIS Pro (version 3.3.0). We dropped a pin for each deer location in ArcGIS and recorded whether the deer was in open or forested landcover. Areas with sparse tree cover were considered open habitat if they were unlikely to block spotlight visibility. Hedgerows were rarely thick enough to block spotlight visibility and were therefore classified as open landcover. We noted whether each deer was standing or bedded. Bedded deer were identified by their lack of whole‐body movement, lower legs not visible beneath the body, and often C‐shaped body orientation. Most often their heads were up but sometimes laid down. Standing deer were easier to positively identify because of feeding or walking movement, or if we gained a clear view of their legs beneath the torso. The bedding behavior of deer that the drone passed directly over (precluding a view of lower legs) and that did not move much could not be determined and was not included in the analysis of bedding behavior. These deer were probably bedded more often than standing, and therefore, we likely undercounted bedded deer by an unknown margin.

We used 30‐m resolution NLCD data (Dewitz, 2021) to determine the distribution of forested and open landcover at each site. We measured the distance to the nearest road for each deer location and for each 30‐m raster cell of our study areas. This enabled us to contextualize the distribution of observed deer with the distribution of sampled and available landcover.

We also used video footage to estimate a green‐up index for each site during each survey. Prior to leaf out, thermal signatures for tree branches were warmer than the surroundings and were sharply delineated on imagery. As spring progressed, thermal signatures around trees and bushes became muddled and hazy because of developing leaves. Trees in open areas were often the first to leaf out, followed by trees on the forest edge, and lastly by trees within the forest interior. For each site, we summed the number of the following conditions that were met to create a green‐up index that ranged from 0 to 6:

1. At least one tree in an open area had visible leaf out.
2. More than half of trees in open areas had visible leaf out.
3. At least one tree on forest edge had visible leaf out.
4. More than half of trees on forest edge had visible leaf out.
5. At least one tree within forest interior had visible leaf out.
6. More than half of trees within forest interior had visible leaf out.

Our focus was on the early stages of vegetative green‐up, so while an index value of 6 represented maximum green‐up during our study, it did not represent full spring leaf out. See Delaney, Harms, and Dinsmore (2025) for video imagery of the range of green‐up conditions during our surveys (“Drone_video_early_season.mp4” and “Drone_video_late_season.mp4” at https://doi.org/10.25380/iastate.30117049.v1).

### Analysis

We processed data in ArcGIS Pro and exported data to R (version 4.4.2) for analysis.

We examined correlations among all potential covariates (Appendix S1: Figure S3). Day of year and green‐up index were highly correlated (*r* = 0.86), so we only used day of year as a predictor. We used site as a random effect in all mixed models described below. Ninety‐five percent of flights were conducted prior to 5.5 h after sunset; one night we recharged batteries and surveyed two additional sites at 7.75 and 8.75 h after sunset. This created outliers for the variable time of night and therefore we focused time of night tests only on data prior to 5.5 h after sunset.

#### Availability bias

To test how day of year covaried with availability bias, we built a generalized linear mixed model and used whether deer were located in the forest or open landcover as a binary response variable. We fit day of year, percent forest, and the number of hours after sunset flights began as site‐level predictors. We also included quadratic terms for each predictor. We removed the number of hours after sunset because it was not a significant predictor and to increase the sample size to construct the final model.

#### Bedding behavior

To assess the factors that affect whether deer were bedded, we built a generalized linear mixed model with bedded or standing as a binary response variable. We used day of year, hours after sunset (site level), and whether deer were in the forest or open landcover (factor; individual level). We also included quadratic terms for day of year and hours after sunset.

#### Distribution from roads

We sampled a greater amount of area closer to roads (Appendix S1: Figure S4) and thus needed to account for variable sampling effort at each distance from roadways. To do this, we calculated a probability density function (PDF) that described the area sampled at each 1‐m wide distance bin (approximated from 30‐m raster resolution [Appendix S1: Figure S4]). Next, we multiplied the PDF of the sampling area by the total number of deer observed to calculate the expected number of deer that would have occurred at each distance if deer were uniformly distributed. We divided the vector of observed deer in each distance bin by the vector of expected deer in each distance bin to express observations as a percent deviance from a uniform distribution. We did this for deer in all habitat types, and separately for deer in the open and deer in forest.

We used two models to test questions related to deer distribution from roads which both used the number of deer observed relative to expected as the response variable. First, we focused on data within the first 200 m of roads to test for avoidance within the distance that a detection function is formed. To do this, we fit a spline using a general additive model (GAM) which allowed maximum flexibility to model nonlinearity over this distance. Second, we focused on the entire data range out to 810 m to test whether spotlight surveys that sample near roads are extrapolatable farther from roads without correction factors. To do this, we fit a linear model with linear, quadratic, and cubic fits of distance as predictors.

## RESULTS

We detected 1043 deer, which averaged 58 deer per hour of flight time and a density of 0.34 deer/ha (assuming perfect detection by drone). Green‐up was restricted to relatively few trees in open areas or forest edges at the start of the study (April 9–13) but increased with the day of the year to a point that most sites had at least 50% of trees with visible leaf out by the end of the study (April 24–30; Appendix S1: Figure S3).

### Availability bias

**FIGURE 1:**
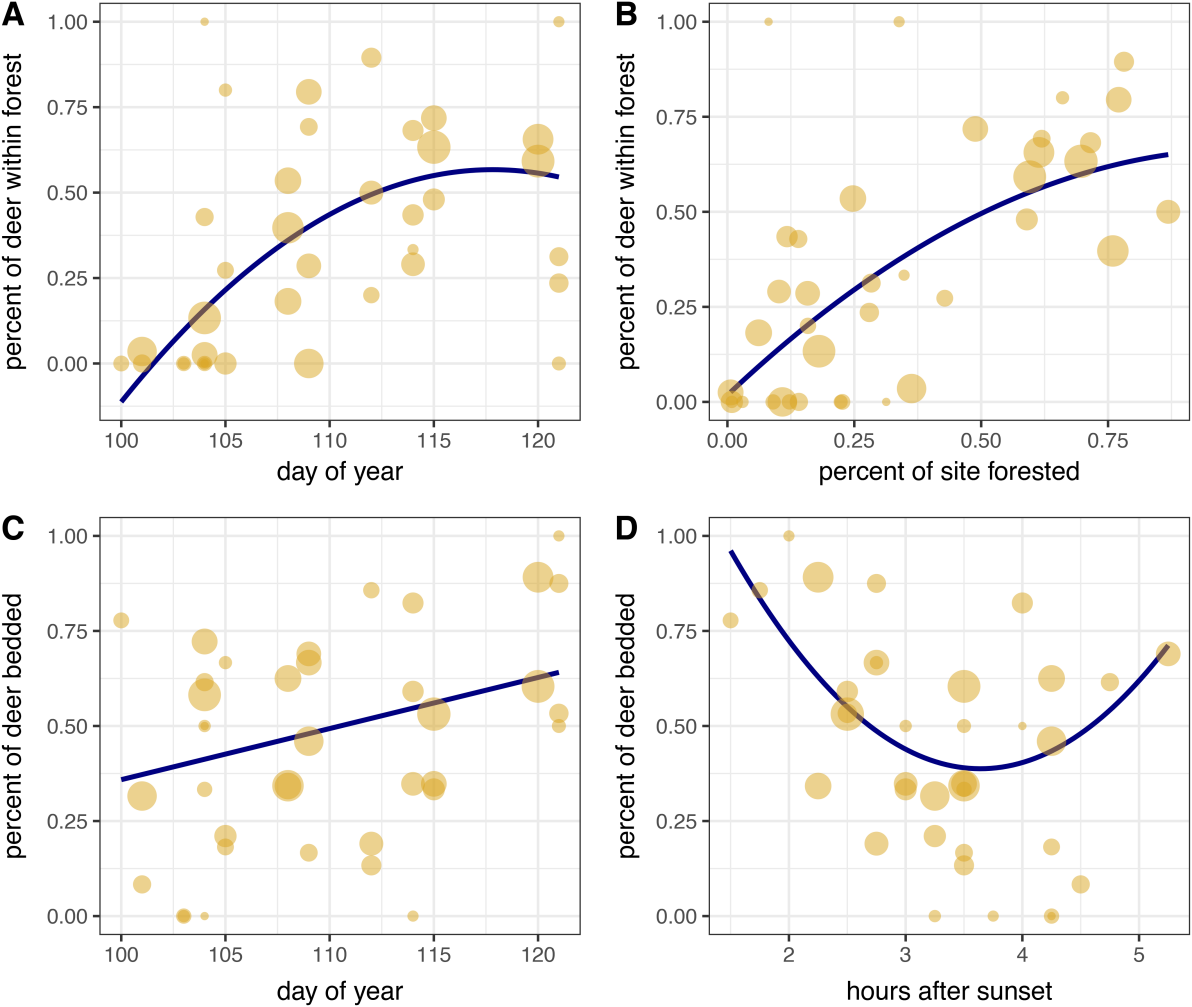
Effects of covariates on the (A and B) percent of white‐tailed deer (*Odocoileus virginianus*) within forest and (C and D) percent of deer bedded during a thermal‐imaging drone study in Iowa, USA, 2024. Each response was analyzed at the individual level with site as a random effect, but each point represents data summarized at the site level. Point size indicates the sample size of deer at each site. Regression lines are plotted over the raw data and weighted by sample size. Statistical results are reported in Table 1.

On average, 38% of deer were located in forest during surveys, but this varied considerably depending on the day of the year and the percent of forest at sites (Table 1). Use of forests was negligible at the start of our study (April 9), increased to approximately 50% by April 23, and then plateaued (Figure 1A). The proportion of deer detected within forest was greater at sites with higher percent forested landcover (Figure 1B). Forest use was not correlated with the time of night (Table 1).

**TABLE 1:** Effects of observation‐ and site‐ level covariates on whether white‐tailed deer (*Odocoileus virginianus*) used forested or open habitat and whether deer were bedded or standing during a thermal‐imaging drone study in Iowa, USA, 2024.

Predictors	Forest use			Bedding		
	Coefficient	Lower 95% CI	Upper 95% CI	Coefficient	Lower 95% CI	Upper 95% CI
Intercept	0.44	−0.37	1.25	**−0.63**	**−1.02**	**−0.27**
Day of year	**0.63**	**0.24**	**1.05**	**0.42**	**0.23**	**0.85**
Day of year^2^	**−0.64**	**−1.06**	**−0.24**	0.05	−0.16	0.39
Time of night	−0.08	−0.47	0.31	−0.26	−0.58	0.01
Time of night^2^	0.20	−0.08	0.47	**0.43**	**0.22**	**0.65**
Percent forest	**0.98**	**0.61**	**1.40**	−0.11	−0.25	0.40
Percent forest^2^	**−0.56**	**−1.15**	**0.00**	−0.19	−0.64	0.25

*Note*: Estimates that were significantly different than zero are bolded.

### Bedding behavior

On average, 50% of deer were bedded and this varied depending on the day of the year and time of night (Table 1). Deer were more likely to be bedded later in April (Figure 1C). The likelihood that deer were bedded decreased until about 3.5 h after sunset and then increased later in the night (Figure 1D). The percent of site forested did not covary with bedding behavior (Table 1).

### Distribution from roads

**FIGURE 2:**
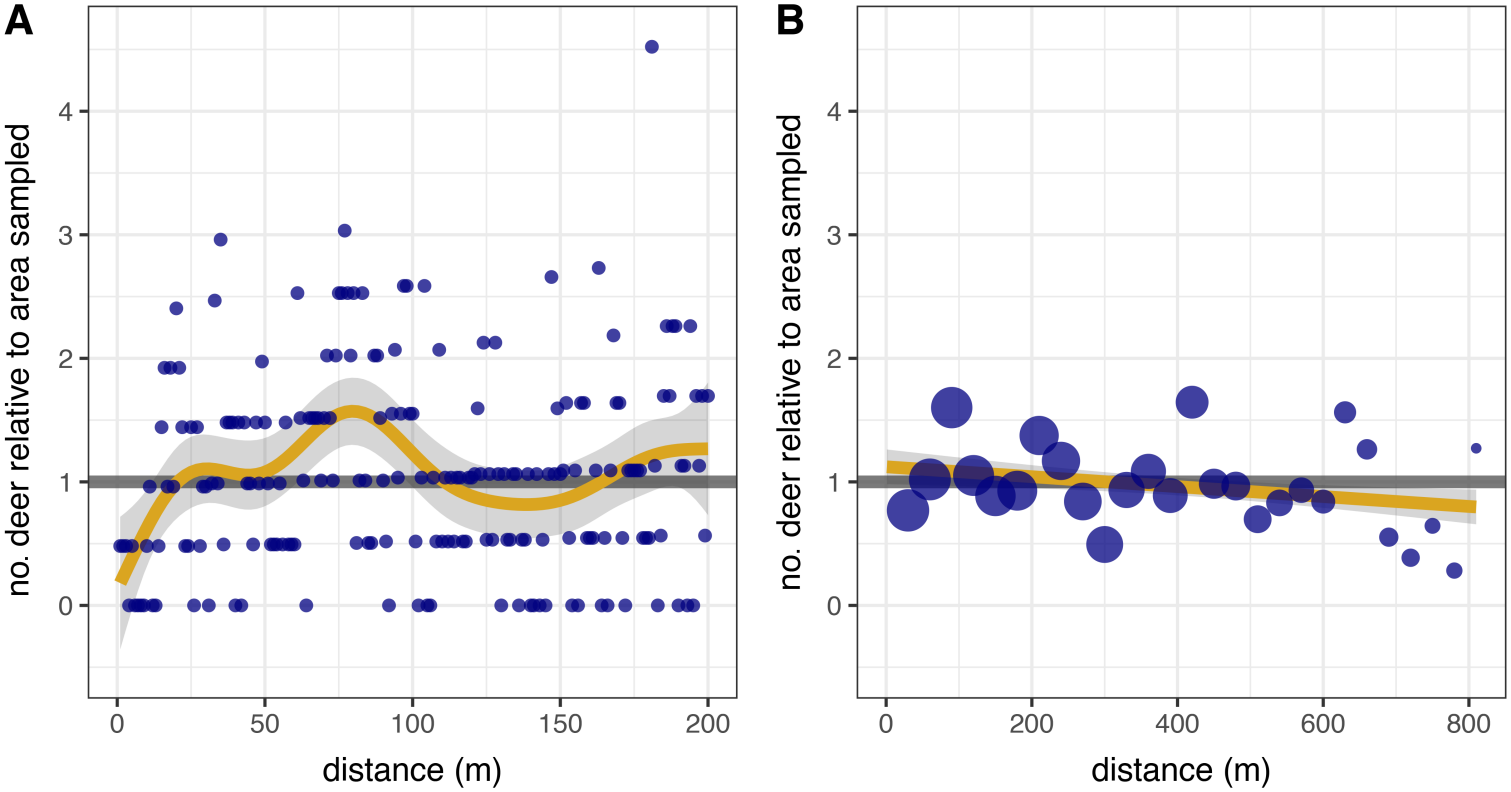
Distances that white‐tailed deer (*Odocoileus virginianus*) were located from gravel roads in Iowa, USA, during a nocturnal drone study in 2024. Each point summarizes the number of deer observed per (A) 1‐m or (B) 30‐m‐wide distance bin divided by the expected number given variable sampling effort at each distance (see *Methods* for details). Thus, values below the horizontal gray line at 1 indicate that less deer were observed than expected, whereas values above 1 indicate that more deer were observed at that distance than expected.

Fewer deer were observed than expected over the first 20 m from roads and were observed more than expected to a peak around 80 m (Figure 2A; edf: 7.0, *F* = 3.5, *p* = 0.001). Over the full data range, the density of deer was rather consistent (Figure 2B; linear estimate [lower 95% CI, upper 95% CI]: −0.0004 [−0.0007, −0.0001]). Neither the quadratic (−8.4e‐7 [−2.3e‐6, 6.0e‐7]) nor cubic (−1.1e‐10 [−7.1e‐9, 6.9e‐9]) distance terms were significant. These patterns were similar for observations restricted to either open or forested landcover (Appendix S1: Figure S5).

## DISCUSSION

Drones have been increasingly used to aid wildlife research, especially to count animals. We explored how a drone study could be conducted to gain confidence and improve the accuracy of density estimates from traditional survey techniques that can be implemented with lower effort across broader spatial extents. Our drone surveys revealed substantial availability bias resulting from forest use, heterogenous bedding behavior (which likely affects detection probability) depending on survey timing, and a nonrandom distribution of deer from transect routes.

### Availability bias

Spotlight survey counts of deer have been criticized as unreliable because of unpredictable variation in detection probability and availability (Collier et al., 2013). We found that forest use was indeed variable, but this variation was well predicted by one temporal and one spatial variable. First, we found that the proportion of deer using forest during surveys increased from nearly 0% to greater than 50% over a 2‐week period in April. This result mirrors a 3‐year study of temporally replicated spotlight surveys that counted fewer deer as spring progressed (Delaney et al., 2025b). We previously posited that fewer deer are counted later in spring because of emerging vegetative growth in forests, which reduces the need to forage in open landcover. Our results here confirm that deer indeed increase their use of forest as spring progresses. Second, forest use increased with the percent of the site that was forested. Because deer density is often higher in areas with more forest cover (Kaminski et al., 2019; Murphy, 1970), availability bias during ground‐based spotlight surveys is likely greater at sites with more deer (Delaney et al., 2025b) as is also the case with viewshed bias (Delaney et al., 2025a). Nevertheless, our study adds to the literature (e.g., Corlatti et al., 2016; Garel et al., 2010) demonstrating that spotlight surveys can be a reliable method for estimating ungulate population size in highly forested regions by sampling when food limitation drives most of the population into open areas to feed.

These results inform future abundance estimation efforts in two ways. First, surveyors can sample when animals are maximally exposed so counts are less affected by low or variable detection rates (e.g., early morning bird counts, Robbins, 1981). Instead, if sampling occurs across a range of temporal or spatial conditions that affect availability, researchers can model those effects on detection probability (for N‐mixture designs) or implement correction factors (for distance sampling designs). N‐mixture studies can model availability covariates on detection probability to improve the accuracy of abundance estimates and the estimated ‘detection probability’ would then be the combination of both perception plus availability probability (Kéry & Schmidt, 2008). For distance sampling, availability bias is confounded with the abundance estimate instead of the detection estimate and therefore would require a correction factor to improve the accuracy of abundance estimates.

### Consequences for detection

Detection probability can be lower for ungulates that are bedded compared to individuals that are standing (Jacques et al., 2014; Zabransky et al., 2016; but see Phillips et al., 2019). Given this, we wanted to test whether the proportion of the population of deer that are bedded changes depending on the timing of surveys. We found that time at two scales affected the proportion of deer bedded. The proportion of deer bedded decreased until about 3.5 h after sunset and then increased until about 6 h after sunset, suggesting deer are probably most detectable around 3.5 h after sunset. Early in the night (~1.5 h after sunset), most deer are probably bedded after having already completed their first foraging bout of the evening (see fig. 3 in Ozoga & Verme, 1970). The increase in standing activity to around 3.5 h after sunset probably represents the next foraging bout followed by an increase in bedding activity again.

The proportion of deer bedded also increased from about 35% to about 65% from the beginning to the end of the sampling season. This is likely because foraging requires more time when food resources are low prior to green‐up. As green‐up progressed and food resources increased, deer probably spent more time bedded because less time was needed to forage. Interestingly, high bedding activity late in the year covaries with lower availability because of increased forest use later in the year. From a survey design standpoint, these findings suggest researchers should sample early in the year prior to vegetative green‐up to maximize animal availability and detectability. If surveys occur across a range of days and times of night, these variables should be modeled on detection probability to reduce the bias of estimates (Duarte et al., 2018; Veech et al., 2016).

### Distribution from roads

Deer have been documented avoiding roads out to 20 m (McShea et al., 2011) and 80 m (Giudice et al., 2019), but in other locations deer density was higher along roads (Beaver et al., 2014; McShea et al., 2011) or in areas of high road density (Munro et al., 2012). We wanted to test whether deer were uniformly distributed from gravel roads at two spatial scales that have the opportunity to bias density estimates in Iowa. First, we found that deer avoided gravel roads out to about 20 m and we saw more deer than expected around 80 m from roads. Perhaps the cluster of deer around 80 m were deer that would have otherwise been within 20 m of roads (i.e., deer that were avoiding roads and clustering around 80 m). Another possibility is that the spike is sampling noise that is similar in magnitude to other apparent stochastic deviations that occurred around 300 and 410 m (Figure 2B). In favor of the prior, we have more data informing the spike at 80 m than farther distances and long‐term spotlight survey data from gravel roads in Iowa (Appendix S1: Figure S1) and Minnesota (Giudice et al., 2019) suggest avoidance extends out to around 80 m. One reason road avoidance may appear more pronounced in historic Iowa DNR spotlight survey data compared to our drone data could be that deer detect spotlight surveyors approaching in vehicles and responsively move farther from roads prior to detection. Giudice et al. (2019) used infrared optics and thought they detected deer prior to responsive movement. During our drone surveys, we parked in strategic locations to not disturb deer and did not observe any traffic during our surveys. Thus, our drone surveys likely recorded deer locations without immediate traffic disturbance whereas the Iowa DNR spotlight survey distributions of deer could include responsive movement to observers or other traffic.

In light of our observed nonuniform distribution of deer from the observer, how should distance sampling of deer in this system proceed? One option is to create a PDF that describes the expected distribution of deer from transects into the distance sampling model structure as has been done when the viewshed is not a traditional rectangle or circle (Delaney et al., 2025a; see also Marques et al., 2013). Another option is to analyze distance as a binned variable and to select a bin width that encompasses the responsive movement (e.g., 100 m). A future drone study that follows spotlight survey vehicles and records deer movements could test whether a distance exists that encompasses all responsive movement.

Roads grid most of Iowa at 1.6‐km intervals, meaning the majority of Iowa exists within 800 m of roads. Given that road‐based spotlight surveys sample deer density within an eyesight range of 300–400 m (Dinsmore et al., 2024; Giudice et al., 2019; Kaminski et al., 2019), we wondered whether estimated spotlight survey densities were representative of non‐sampleable area > 400 m from roads. At this larger scale, we found a very slight decline in deer density with distance from roads that may result from a combination of stochasticity and inadequate sample area informing longer distances. Regardless, the slightness of this effect suggests spotlight survey densities likely provide a reasonable measure of density farther from roads in Iowa.

### Future drone study in Iowa

We suggested that researchers using spotlight surveys for deer conduct surveys early in the spring prior to green‐up to maximize availability and detection probability. The present study began on April 9, just as vegetation was emerging. Future drone surveys should be conducted in March and early April to confirm that availability and detection are also high during the months leading up to vegetation emergence. Multiple years of this work may also enable the separation of variation caused by survey date versus green‐up phenology.

Because of the order that we surveyed sites, a correlation existed between survey date and the percent of the site that was forested (*r* = 0.36; Appendix S1: Figure S3). We fit each of these variables as predictors within the same models and still uncovered significant explanatory power for both on availability, marginal to the effect of the other variable. Only survey date affected bedding behavior and models that excluded survey date also estimated no significant effect of percent forest landcover for either linear or quadratic terms (both *p* > 0.71). Future work should focus on site selection that reduces correlation between these variables to better characterize which predictors are inducing variation in availability and bedding behavior.

Lastly, we chose flight parameters, mainly flight height and speed, that we felt maximized detection probability for deer. Nevertheless, we cannot know whether these parameters actually produced perfect detection. Imperfect detection could also result from video processing procedures, such as human fatigue during video processing (possible in our study). Future work that analyzes similar drone footage in a distance sampling design (testing for a decline in detections with distance from center frame) or surveys radio‐tagged deer would provide insight into the detection probability of our drone survey protocol.

### Perspectives and applications

N‐mixture and distance sampling techniques estimate population parameters (i.e., detection probability and abundance/density) and their statistical uncertainties given that system assumptions are perfectly met. Considering this, we suggest that there are three main benefits for assessing system assumptions. First, if researchers are not confident in the validity of system assumptions, then realized uncertainty is greater than the statistical uncertainty estimated by these methods. If these assumptions are validated, then researchers can be confident that the estimated statistical uncertainty represents appropriate bounds. Second, if deviations in assumptions are quantified, researchers can build customized models tailored to their system (e.g., Delaney et al., 2025a; Marques et al., 2013) or apply correction factors post hoc to reduce bias (e.g., Marsh & Sinclair, 1989; Thomson et al., 2013). Third, information gained can be applied to future survey design protocols. As exemplified here, future surveys can be conducted during a time of the annual cycle when availability bias is lowest and detection probability is highest.

Drones are highly effective at detecting and counting animals, but in the near future, ground‐based techniques are likely to remain the primary survey method when density estimates are needed across large spatial scales. Still, we suggest information gained from strategic drone studies can vastly improve the accuracy of estimates from ground‐based surveys. Our drone study revealed that white‐tailed deer availability and likely detection probability (via bedding behavior) shift as functions of day of year, time of night, and landcover, which informs future survey protocols and model structure. Our study was also able to quantify the magnitude of road avoidance which will inform decisions during future distance sampling analysis and extrapolation. More broadly, we show how drone surveys can be used to assess the assumptions of N‐mixture and distance sampling study designs to gain confidence and improve the accuracy of population estimates from ground‐based surveys. Similar use of these emerging technologies is likely to improve density estimation techniques in diverse animal systems.

## AUTHOR CONTRIBUTIONS

All authors designed the study. David M. Delaney collected the data, conducted the analysis, and wrote the first draft. All authors revised and approved the manuscript for submission.

## CONFLICT OF INTEREST STATEMENT

The authors declare no conflicts of interest.

## Supporting information


Appendix S1.

## Data Availability

Data, code, and video footage (Delaney, Harms, & Dinsmore, 2025) are available in Iowa State University's data repository at https://doi.org/10.25380/iastate.30117049.v1.
