# Supplementary material for: Method matters: Use of thermal‐imaging drones to assess the assumptions of density estimation techniques
Source: Ecol Appl. 2025 Dec 8;35(8):e70164. doi: 10.1002/eap.70164 (PMC12686606; doi:10.1002/eap.70164)
Supplement: Supplementary file 1 — Appendix S1. [file EAP-35-e70164-s001.pdf]

**Appendix S1**

# Method matters: Use of thermal-imaging drones to assess the assumptions of density estimation techniques

David M. Delaney, Tyler M. Harms, Stephen J. Dinsmore

*Ecological Applications*

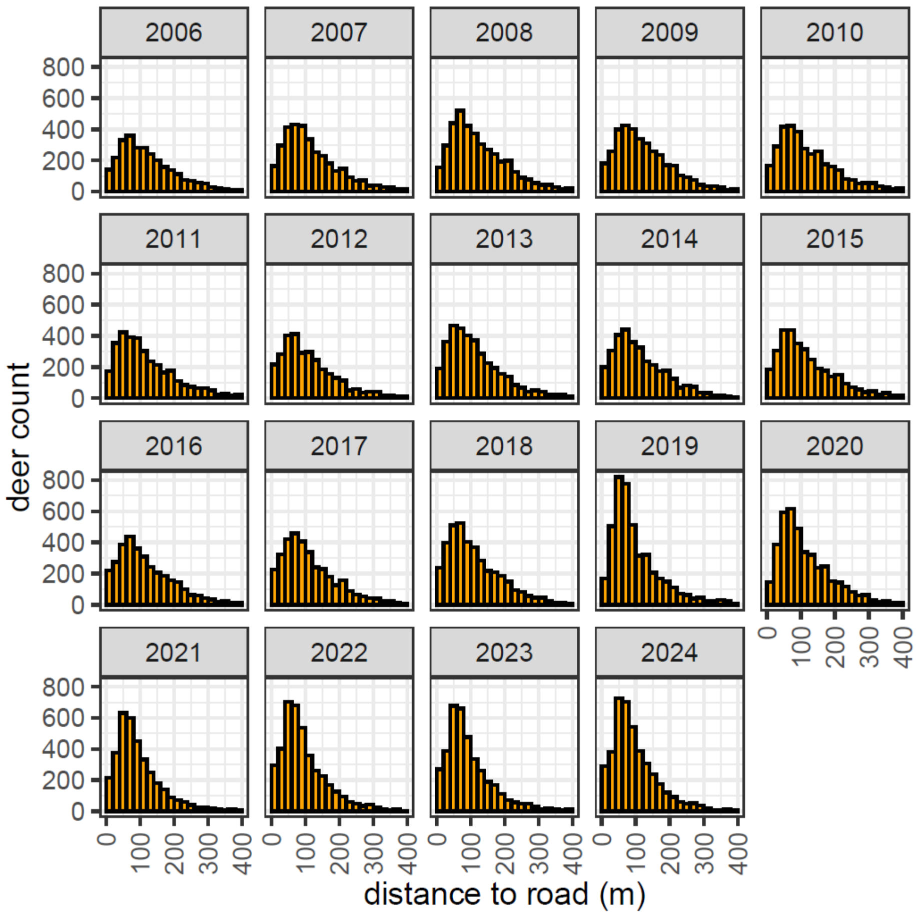


**Figure S1**. Distributions of the distances that white-tailed deer (*Odocoileus virginianus*) were detected from gravel roads in Iowa, USA during nocturnal spotlight surveys. Global Positioning System coordinates were estimated for each detected deer location using a laser rangefinder from 2006–2018 and satellite imagery via a smartphone application (FieldMaps) from 2019–2024. Each distance bin was 20m wide and the peak bin in most years was 60-80m.

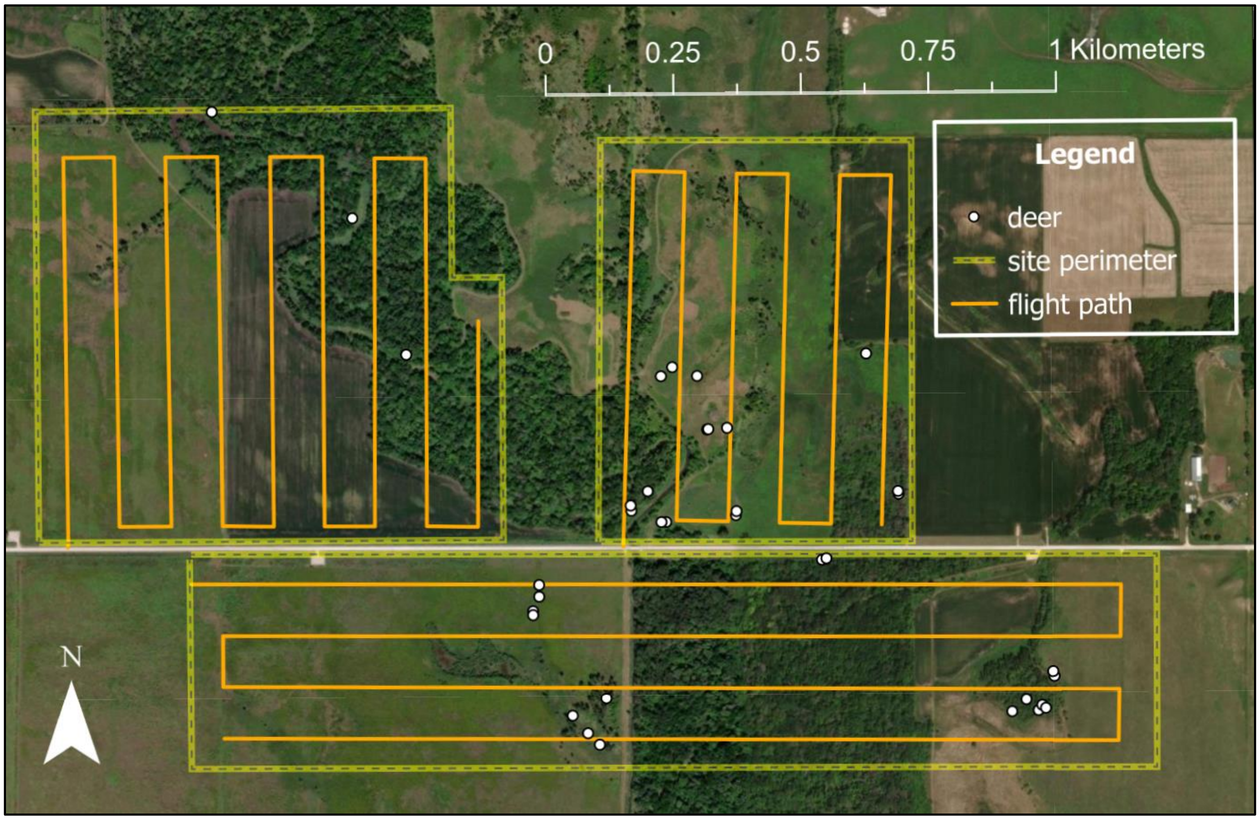


**Figure S2**. Map of three of the study sites. Each site contained a mix of forested and open canopy landcover and occurred adjacent to a gravel road. The gravel road for these sites ran East to West.

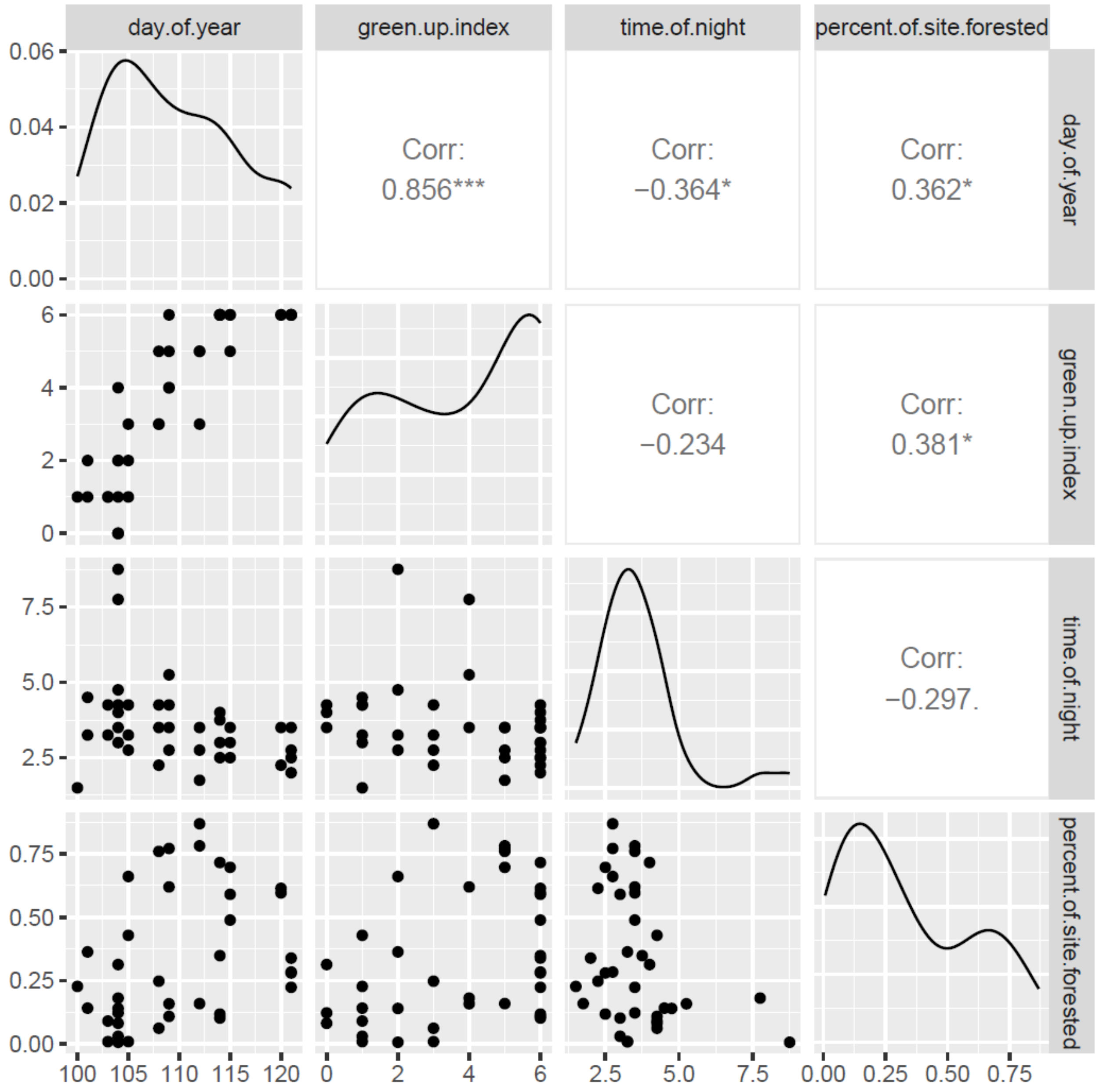


**Figure S3**. Correlations among potential predictors from a thermal-imaging drone study of white-tailed deer (*Odocoileus virginianus*) landscape use and behavior in Iowa, USA, 2024.

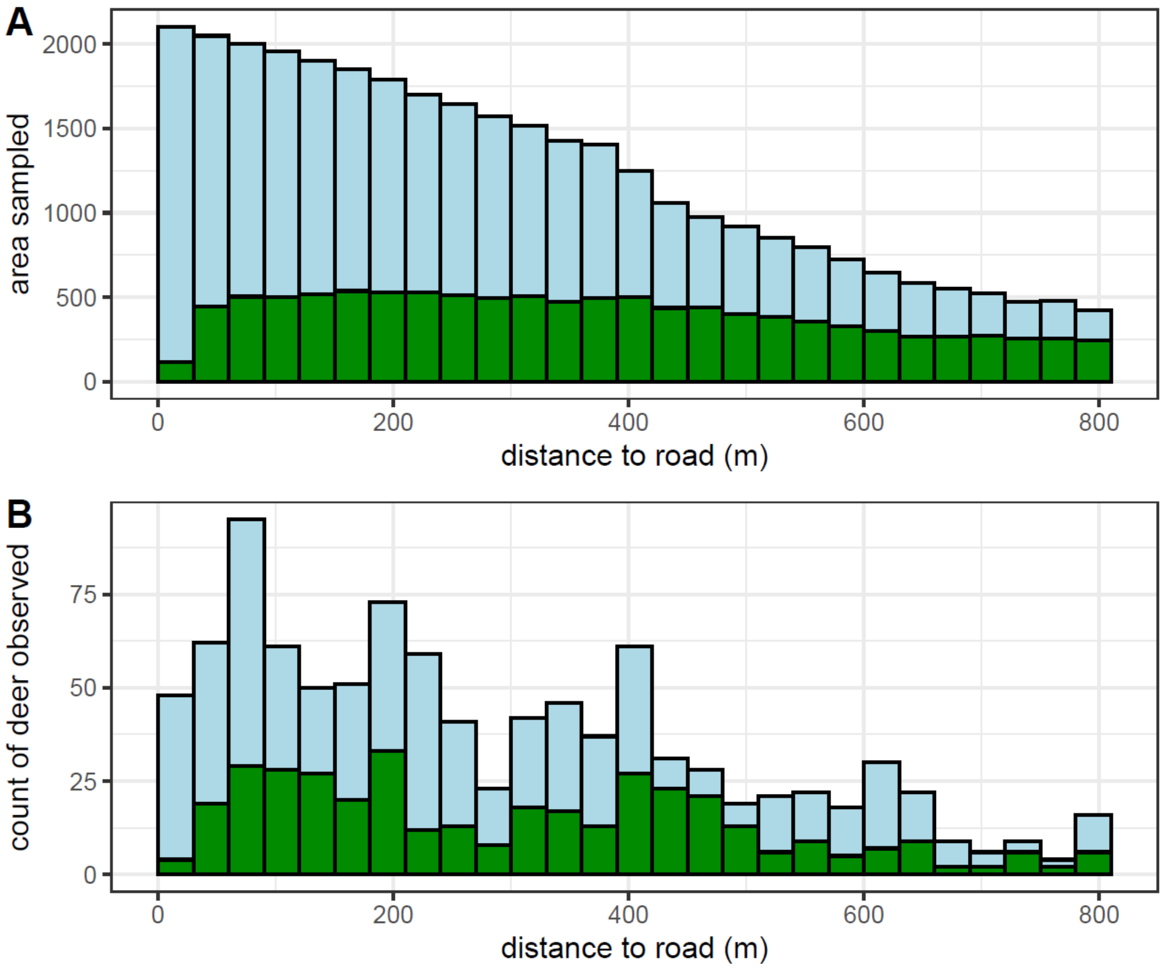


**Figure S4**. Distributions of A) area sampled and B) observed white-tailed deer (*Odocoileus virginianus*) from gravel roads during a nocturnal drone study in Iowa, USA, 2024. Scale of area sampled is the number of 30 $m^2$ raster cells. Green indicates the A) raster cell or B) deer were in forest whereas blue indicates open landcover.

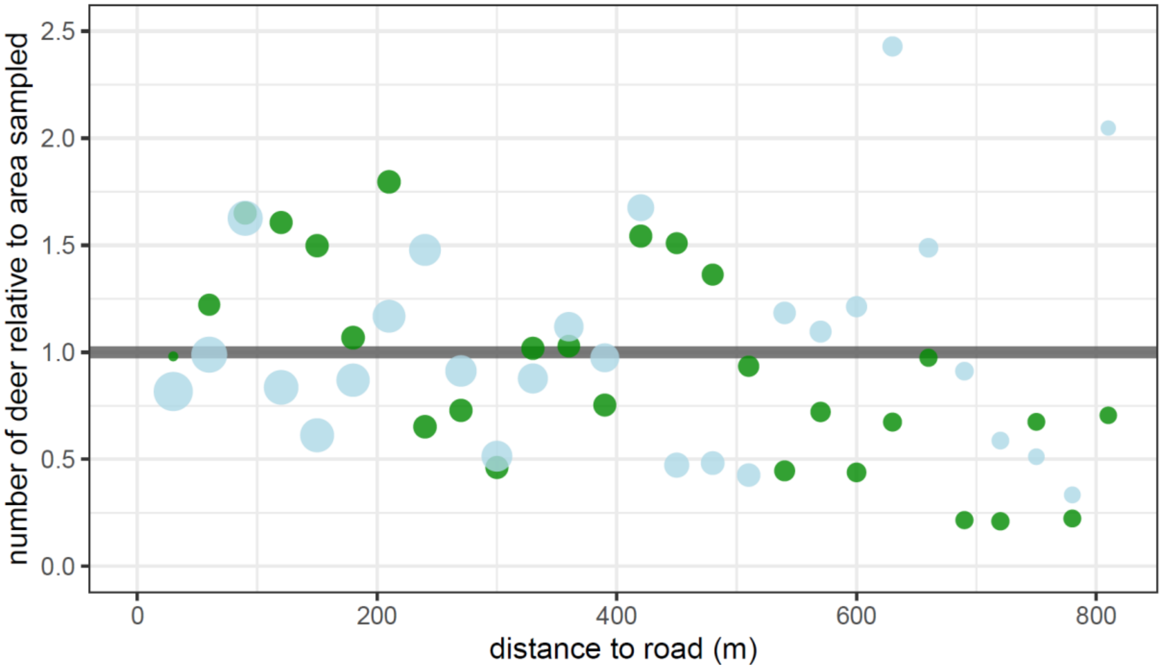


**Figure S5**. Distances that white-tailed deer (*Odocoileus virginianus*) were located from gravel roads in Iowa, USA during a nocturnal drone study in 2024. Each point summarizes the number of deer observed per 30-m wide distance bin divided by the expected number given variable sampling effort at each distance (see methods for details). Thus, values below the horizontal grey line at 1 indicate that less deer were observed than expected whereas values above 1 indicate that more deer were observed at that distance than expected. This distribution was calculated separately for deer in open (blue) and forested (green) landcovers.
